# The incremental value of tuberculosis detecting African giant pouched rats over Smear microscopy and Xpert MTB/RIF for Tanzanian TB detection

**DOI:** 10.1371/journal.pone.0321866

**Published:** 2025-04-29

**Authors:** Tefera B. Agizew, Joseph Soka, Stephen Mwimanzi, Cynthia D. Fast, Gilbert Mwesiga, Nashon Edward, Marygiven Stephen, Rehema Kondo, Christophe Cox, Negussie Beyene

**Affiliations:** 1 Anti-Persoonsmijnen Ontmijnende Product Ontwikkeling (APOPO) Tuberculosis Department, Sokoine University of Agriculture, Morogoro, Tanzania; 2 Department of Biology, University of Antwerp, Belgium; 3 APOPO Tuberculosis Research Project, Armauer Hansen Research Institute, Addis Ababa, Ethiopia; Elizabeth Glaser Pediatric AIDS Foundation, TANZANIA, UNITED REPUBLIC OF

## Abstract

**Background:**

Prior studies that used Anti-Persoonsmijnen Ontmijnende Product Ontwikkeling (APOPO)-trained African giant pouched rats reported an annual average of 40% increase in sputum-smear-positive tuberculosis (TB) detection. Sputum-smear microscopy (smear) is being replaced by Xpert MTB/RIF (Xpert)-based diagnostic algorithms in many countries. We evaluated the incremental values of rat-based case detection over smear and Xpert.

**Methods:**

From January to December 2023, sputum samples were collected from presumptive TB patients at 69 health facilities that use Xpert or smear-based diagnostic algorithms. An average of five rats were used at APOPO to re-evaluate the second sample from Xpert-negative patients and the smear-negative sample. Concentrated smears and Ziehl-Neelsen staining were used to confirm samples that were rat-positive (samples indicated positive by  ≥ 1 rat). We analyzed the increase in case detection (incremental yield) by the rats over smear and Xpert and compared the rise against *Mycobacterium* bacillary load using the Chi-square test.

**Results:**

A total of 43,153 samples from 34,565 patients were collected and re-evaluated by rats. There were 6,717 bacteriologically confirmed TB cases; 4,541 (68%) of these cases were detected at health facilities (3,239 from Xpert and 1,302 from smear sites); the remaining 2,176 (32%) were found by trained rats, representing 48% (2,176/4,541) overall incremental value. Of which, 65% (1,409/2,176) and 35% (767/2,176) were among Xpert and smear-negative patients, respectively. The Incremental yield by rats at Xpert facilities was 44% (1,409/3,239), while it was 59% (767/1,302) at smear facilities, Odds Ratio, OR = 0.74, 95% confidence interval, CI:0.66–0.82. The rats were 47% more likely to identify TB among Acid Fast Bacilli smear 1 +  or scanty from Xpert-negative samples than smear-negative [91% (1,276/1,409) versus 87% (665/767), OR = 1.47, 95% CI:1.12–1.94]. The difference between children and adults, however, was not substantial, [69% (68/98) versus 67% (1,138/1,701), OR = 1.12, 95% CI:0.72–1.74].

**Conclusions:**

Our finding indicated that trained rats reasonably added benefit over DOT TB case findings. According to our data, switching from smear to Xpert sites lowered the added value by more than a quarter, even though the Xpert sites were likely to benefit more case finding among TB patients with lower bacillary loads.

## Introduction

Tuberculosis (TB) is a curable and preventable disease and yet, globally, TB remains a public health concern. Tanzania is one the 30 high TB burden countries [1]. Though there has been a steady decline in TB incidence over the past five years, from an estimated incidence rate of 252 to 195 per 100,000 population, which is an annual average of 5%, TB is still a public health problem in Tanzania [1]. This reduction is far less than the rate needed to end the TB epidemic and meet the ENDTB target [2]. Close to 25% of TB cases are estimated to be missing every year in the country [1]. Thus, accelerated TB case detection using cheaper and sustainable diagnostic tests to find TB patients is essential.

Anti-Persoonsmijnen Ontmijnende Product Ontwikkeling (APOPO), a Belgian nonprofit research organization headquartered in Morogoro, Tanzania trains rats to detect diseases by sniffing biological samples, including sputum. APOPO, a pioneer and the only organization in the world that uses an innovative rat-based TB detection technology, utilizes African giant pouched rats (*Cricetomys ansorgei*) for this purpose [3].

In 2009, APOPO reported for the first time that rats can detect TB [4]. Our subsequent studies revealed that the rats are detecting a bouquet of Volatile Organic Compounds present in the patient sputum samples [5,6]. A recent meta-analysis found that the patient-wise sensitivity and specificity of the rats is 81.3% and 73.4%, respectively [7]. Furthermore, trained rats have high sample throughput (100 samples in less than 20 minutes) and provide cost-effective second-line TB screening especially in high-burden and high transmission probability settings [3]. While further refining the method, it has been implemented as a tool to find missed TB cases in Tanzania as a result of which, by the end of 2023, we found more than 20,000 patients that were missed by the conventional diagnostic method [3].

Prior studies that used APOPO-trained African giant pouched rats reported an average annual increase of 40% (incremental value) over sputum-smear microscopy (smear)-positive TB detection in Tanzania [3,5,8]. Currently, smear is being replaced by Xpert MTB/RIF (Xpert)-based diagnostic algorithms in Tanzania.

While we reported rats value over sputum-smear microscopy, there is limited data on the added value (incremental value defined as TB detected by rat-based evaluation divided by TB detected at Directly Observed Therapy (DOT) facilities) of trained rats over the WHO-recommended rapid molecular diagnostics [9]. All the previous studies were focused on comparing rats against smears [3,5,8]. The aims of the present study were to evaluate: (1) the incremental value of rat-based TB case detection over Xpert as used at health facilities; (2) whether the incremental value of rat-based TB case detection over Xpert has similar benefits as in smear settings; and (3) whether rat-based TB case detection differs by *Mycobacterium* TB bacillary loads and between children and adults. This report has a potential impact for the National TB control program control activities, particularly to the bat-based evaluation if beneficial to DOTs sites where Xpert is an initial diagnostic testing.

## Methods

### Study settings and designs

Our study was a prospective study where presumptive TB patients were recruited as part of routine National TB control activities for the whole calendar year, from January 1^st^ to December 31^st^, 2023, with some follow-ups. Following a first-line TB screening test at DOT facilities, sputum samples were collected for a second-line TB screening by African giant rats. When the test results were confirmed as microbiologically confirmed TB after rats’ indications as TB positive, patients were recalled by our community volunteers for initiation of anti-TB treatment and were followed up through treatment completions.

The study settings were urban and semi-urban health facilities at hospitals or primary health care, 58 of those DOTs centers were at Dar-es-Salaam and around, and 11 at Dodoma, the capital city of Tanzania.

### Study population

The study population included all individuals of all ages, including children, men, and women, who presented with symptoms of TB at the study sites.

### Recruitment and tuberculosis screening

As mentioned above in the study population, when patients of all age group presented for any health care at the study facilities, they were screened for TB by the public health care workers and those presumed to be TB were recruited and provided sputum for initial diagnostic test, smear or Xpert depending on the availability. Children (0–14 years old) and adults (15 years and older, hereafter referred to as adults) were screened per the national TB guidelines. Adults were screened for four TB symptoms (cough, fever, night sweats, and weight loss) for two or more weeks and children were screened for weight loss or failure to thrive (no weight gain > 3 months), cough for ≥ 2 weeks, fever for ≥ 2 weeks, fatigue or reduced playfulness for ≥ 2 weeks, and profuse night sweats for ≥ 2 weeks [10]. Presumptive TB was defined when patients were screened positive for one or more of the TB symptoms using the above criteria.

### Laboratory procedure and APOPO tuberculosis detection model using rats

The operational context of APOPO’s model used “second-line TB screening by rats among presumptive TB patients deemed as negative by routine DOT facilities TB screening and diagnostics. Two spot sputum samples, in case of smear center, and one spot, in case of Xpert sites, were collected and tested the same day using routine Ziehl-Neelsen (ZN) smear-microscopy or Xpert at the DOTS center laboratory. Each day all smear-negative samples, and the second sample from Xpert-negative patients were collected from DOT facilities and transported to the APOPO laboratory by the study sample transporters.

At APOPO the samples were subjected to heat-inactivation at 90^0^C for 30 min before presenting the samples to rat’s evaluation. Five rats on average sequentially evaluated the samples placed under 10 sniffing holes in the floor of a rectangular chamber (205 cm long, 55 cm wide, and 55 cm high). A positive rat response (‘indication’) was taught through operant conditioning during training and was defined as the rat holding the nose in the scent hole for three seconds or more. Rat-trainer (also called handlers) observed the rat as it moved along the line of holes sniffing the samples and recording all positive indications. After rat evaluation, rat-indicated (rat-positive) samples were centrifuged and analyzed at the APOPO laboratory using concentrated ZN smear-microscopy [4,8].

**Fig 1:**
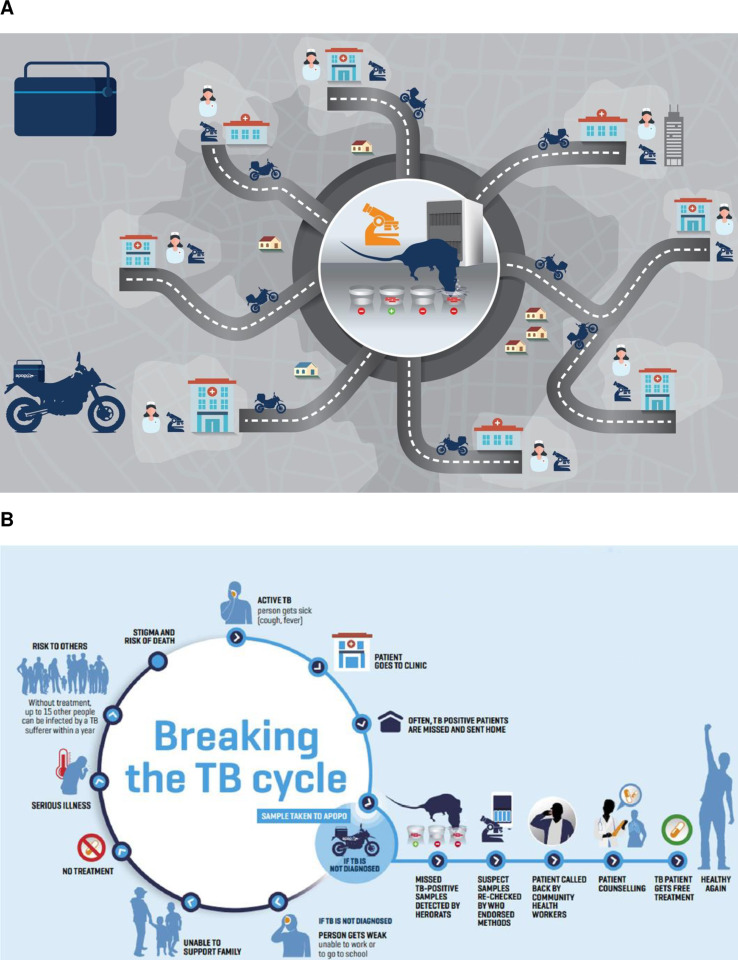
A. APOPO’s Hub and Spoke sample transportation model, B. APOPO tuberculosis detection model using rats. **Reproduced with permission** [11].

The National TB and Leprosy Program-approved reporting form was used to report patients with rat-positive samples that were verified by concentrated ZN back to the DOT facilities. Then, the community volunteers partnering with APOPO recalled the patients back to the health facilities and made it possible for them to receive anti-TB therapy (Fig 1) [3,11,12].

### Data collection

Data were collected using standardized case report forms (CRF) between January and December 2023 at the DOT facilities when patients presented for health care and at APOPO laboratory following rat’s assessment. Age, gender, names of health facility, initial diagnostic tests at DOT facilities, *mycobacterium* bacillary loads and confirmatory tests at APOPO laboratory and the final test results at APOPO were main variables of interest. An in-house-developed TB Laboratory Information System (TB-LIMS) was used to record the laboratory related data such as rat’s assessment and results, and confirmatory diagnostic tests performed and its results before sending the result to the DOT facilities. Logic checks were used to find inconsistencies, which were then verified against the original CRF. Consistencies and missing data were, whenever possible, fixed by reviewing patient charts. Our data collection method was described previously [9].

### Statistical analysis

Data were analyzed using STATA statistical software 15 [13]. To address our main objectives, comparing rats versus smear, rats versus Xpert, the main analysis was over incremental value, which is the number of TB detected by rat-based evaluation divided by TB detected at DOT facilities. We also calculated the proportion of TB detected by rat-based evaluation over the total TB reported at the DOT facilities. A Chi-square test was used to assess the incremental values over Xpert MTB/RIF and smear, *Mycobacterium* TB bacillary load among children and adults, as well as the demographic and laboratory parameters of the patients. We employed an odds ratio and a 95% confidence interval, and statistical significance was determined by *P values* less than 0.05.

### Ethical considerations

The study protocol was approved by the Institutional Review Board (IRB, Ref. nr. NMRI/HQ/R.8c/Vov.I/2503) at the Tanzanian National Institute for Medical Research (Dar es Salaam, Tanzania). Following IRB approval, patients were enrolled in the study, and data were collected as part of the national TB program’s routine TB diagnostic care activities. Therefore, the ethical committee waived the need for informed consent for this operational research that involved second-line TB screening following routine care.

Pertaining to the rats, all procedures were conducted in accordance with all relevant guidelines and regulations of the Sokoine University of Agriculture. The methods and results reported herein adhere to the National Centre for Replacement Refinement & Reduction of Animals in Research ARRIVE 2.0 guidelines. At the conclusion of all experiments, subjects remained housed with no animals sacrificed.

### Results

From the 34,565 patients screened, a total of 43,153 samples were collected and evaluated by rats between January and December 2023. Children and adults were 6% (2,014/32,228) and 94% (30,214/32,227), respectively. The median age was 39 years (interquartile range, 27–51 years), and 59% (20,284/34,505) were males ().

**Table 1:** Demographic characteristics of presumptive tuberculosis patients screened at health facilities in Tanzania.

Characteristic	N	Percentage/median
Patients	34,565	100%
Age		
Median (IQR)		39 (27–51)
<15 years	2,014	6%
≥15 years	30,213	94%
Missing	2,338	
Gender		
Male	20,283	59%
Female	14,221	41%
Missing	61	

IQR = interquartile range

### TB detection using rats

**Fig 2:**
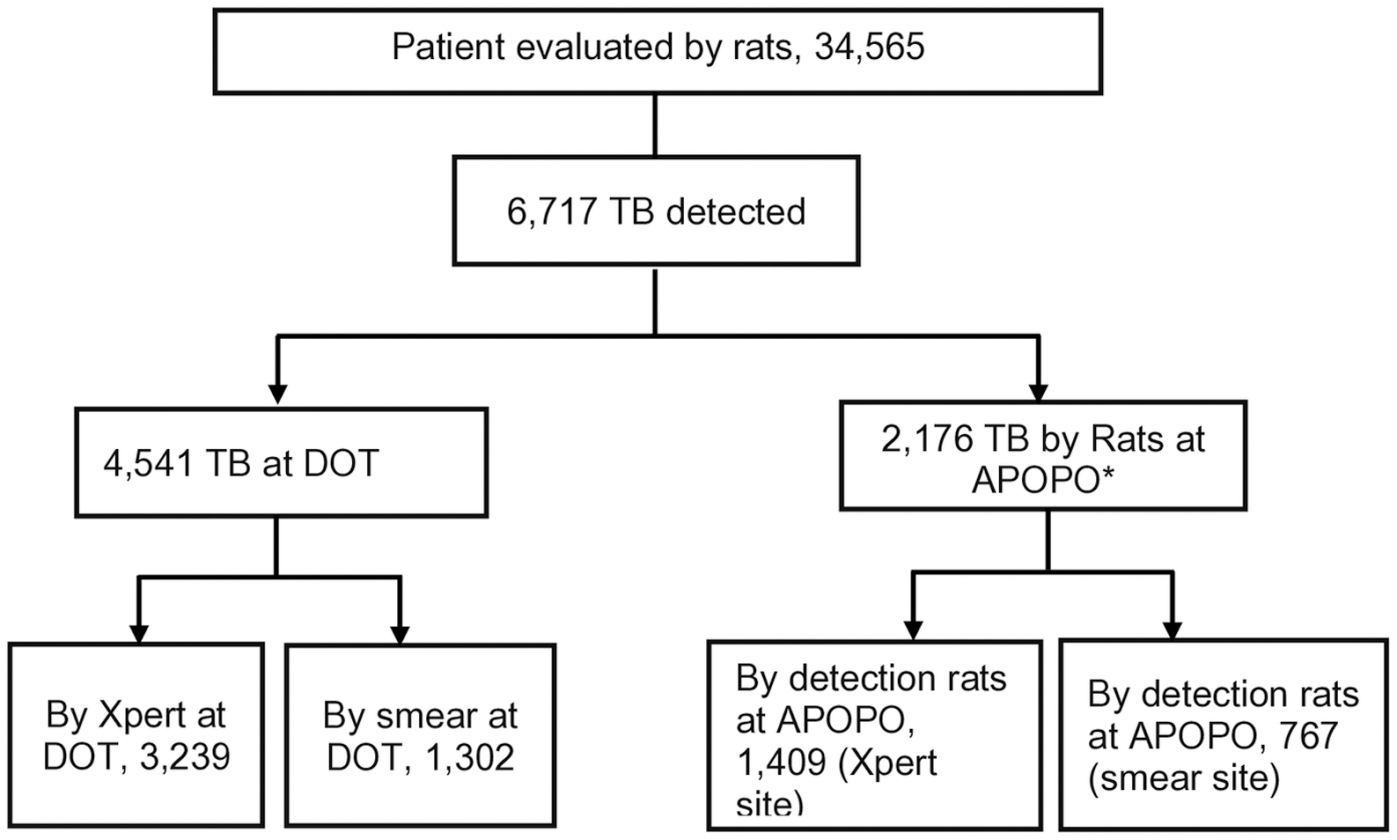
Presumptive tuberculosis and bacteriologically confirmed tuberculosis cases in Tanzania study sites from January to December 2023. **Note:** * *By detection rats at APOPO and confirmed by concentrated ZN sputum smear microscopy.*

**Fig 3:**
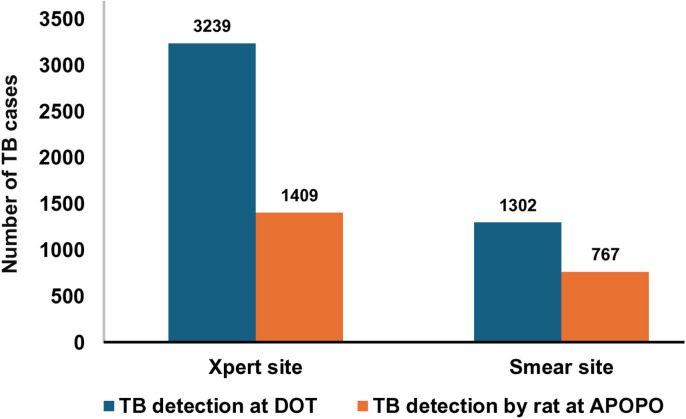
Incremental value of rat-based TB detection between sites using Xpert and smear-based diagnostic algorithm in Tanzania study sites from January to December 2023.

Of the 6,717 TB cases, 4,541 (68%) were detected at DOT facilities using Xpert testing (3,239) or smear (1,302). Trained-rats detection contributed to 2,176 (32%) of the TB cases, yielding an overall 48% (2,176/4,541) incremental value. Among TB cases detected by rats at APOPO, 65% (1,409/2,176) and 35% (767/2,176) were among Xpert and smear-negative patients, respectively. The incremental yield by rats at Xpert facilities was 44% (1,409/3,239), while in smear facilities, it was 59% (767/1,302), Odds Ratio, OR = 0.74, 95% confidence interval, CI:0.66–0.82, (Fig 2 and 3).

### TB detection using rats among children and adults by *Mycobacterium* bacillary load between Xpert and smear sites

Trained-rats were 47% more likely to identify TB among Acid Fast Bacilli smear 1 + or scanty from Xpert-negative patients than smear-negative patients [90% (1,267/1,409) versus 87% (665/767), OR = 1.47, 95% CI:1.12–1.94] (Table 2). The difference between children and adults, however, was not substantial, [69% (68/98) versus 67% (1,138/1,701), OR = 1.12, 95% CI:0.72–1.74] (Table 3).

**Table 2:** Characteristics of tuberculosis patients detected by rats at APOPO among patients with Xpert MTB/RIF negative and sputum-smear microscopy negative at DOT facilities in Tanzania.

Characteristic	N	TB by detection rats at APOPO^*^ from Xpert sites, n = 1,409	TB by detection rats at APOPO* from smear sites, n = 767	OR	95% CI	p-value
		n (%)	n (%)			
Age						
<15 years	109	73 (67)	36 (33)	1.05	0.70–1.58	0.821
≥15 years	1,913	1,261 (66)	652 (34)			
Gender						
Male	1,303	854 (66)	449 (34)	1.08	0.90–1.29	0.401
Female	867	553 (64)	314 (36)			
Bacillary load (AFB)						
Scanty	1,522	1,019 (67)	503 (33)	**1.47** ^**^	**1.12–1.94**	**<0.006**
1+	419	257 (61)	162 (39)			
2+	173	97 (56)	76 (44)			
3+	62	36 (58)	26 (42)			

* TB at APOPO lab were Xpert MTB/RIF (Xpert) or Acid-Fast Bacilli (AFB) negative at DOT facilities, then re-evaluated by rats and confirmed later using concentrated Ziehl-Neelsen (ZN) smear microscopy.

** The Odds ratio was calculated by combining scanty and AFB 1 + together and AFB 2 + and 3 + together APOPO = Anti-Persoonsmijnen Ontmijnende Product Ontwikkeling. DOT = Directly Observed Therapy

**Table 3:** Tuberculosis patients evaluated using rats at APOPO among Xpert MTB/RIF and smear negative at DOTs facility by *Mycobacterium* bacillary load between children and adults in Tanzania.

Characteristic	N	Children	Adult	OR	95% CI	p-value
		n (%)	n (%)			
1 + or Scanty by detection rat from Xpert DOT site	1,206	68 (6)	1,138 (94)	1.12	0.72–1.74	0.612
1 + or Scanty by detection rat from smear DOT site	593	30 (5)	563 (95)			
Total	**1,799**	**98**	**1,701**			

* Acid Fast Bacilli (AFB)

**Fig 4:**
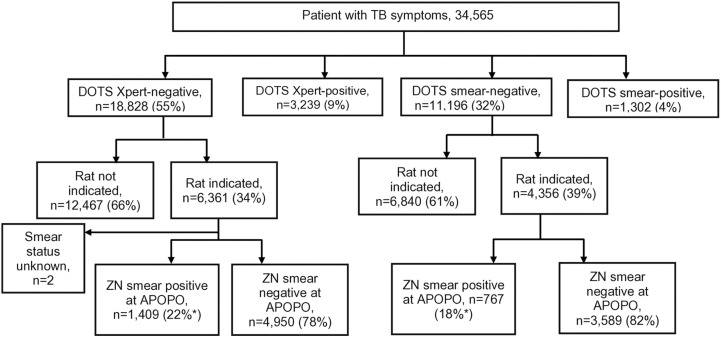
Rat indication and tuberculosis cases after ZN smear confirmation among presumptive tuberculosis patients in Tanzania study sites from January to December 2023. **Note:** * Positive by detection rats at APOPO and confirmed by concentrated Ziehl-Neelsen (ZN*) sputum smear microscopy. Since the samples were not tested by culture, 22% and 18% for Xpert group and smear group do not truly represent sensitivity, since the* ZN *smear is not good enough in sensitivity, nor does 78% for Xpert and 82% for smear group represent a true false positive by rats’ evaluation.*

Rats-based TB detection method was 10% more likely to detect TB **among presumptive TB patients** with Xpert negative versus smear negative result: 7% (767/11,196) versus 8% (1,409/18,282), OR = 1.10, 95% confidence interval: 1.01–1.20, *p value = *0.04 (Fig 4)*.*

### Performance of rats against TB cases with microbiological evidence by smear or Xpert

Among sputum samples that we use for rewarding purposes, rats have re-evaluated 1300 smear-positive and 1166 Xpert-positive TB patients from DOT facilities. The trained rats’ sensitivity and specificity against smear and Xpert were 82.3% versus 98.7% and 65.6% versus 71.6%, respectively. Rats were 16 times more likely to identify TB in patients who tested positive using Xpert than in those who tested positive by smear. [98.7% (1,151/1166) versus 82% (1070/1300), OR = 16.47, 95% CI: 95% CI: 9.78–27.83], *p value* <0.001. And 1.3 times more likely to rule out TB among presumed TB patients tested negative by Xpert than using smear, [71.6% (12467/17417) versus 65.6% (6,840/10,429)), OR = 1.32, 95% CI:1.25–1.39] *p value* <0.001 (Table 4).

**Table 4:** Sensitivity, specificity and predictive values of rats compared with smear- and Xpert-positive.

	Sensitivity % (95% CI)	Specificity % (95% CI)	PPV % (95% CI)	NPV % (95% CI)	Sensitivity % (95% CI)	Specificity % (95% CI)	PPV % (95% CI)	NPV % (95% CI)
	82.3 (80.1-84.3)	65.6 (64.7-66.5)	23.0 (21.8-24.2)	96,7 (96.3-97.1)	98.7 (97.9-99.3)	71.6 (70.9-72.2)	18.7 (17.7-19.8)	99.8 (99.8-99.9)
Rat indication	Smear-positive at DOT	Smear-negative at DOT			Xpert-positive at DOT	Xpert-negative at DOT		
Positive	1070	3589	4659		1151	4950	6161	
negative	230	6840	7070		15	12467	12482	
Total	1300	10429			1166	17417		
Not evaluated	2*				2073*			
Grand total	1302				3239			

Note:

* Two of the 1302 Smear positive and 2073 of 3239 Xpert positive at DOT were not used for rat’s rewarding purposes, thus were not re-evaluated by rats.

## Discussions

Close to two-thirds of patients in this study were from DOT facilities, which have shifted to WHO-recommended rapid molecular testing, and the remaining one-third were from sites using smears as initial diagnostic tests. In such smear-diagnostic settings, for more than a decade, trained African giant pouched rats have been used to re-evaluate sputum samples tested by smear under routine program conditions [3,8]. It was previously reported that using trained rats, results in an annual average of 40% increase (incremental yield) in smear-positive case detection over smear [8]. Mgode *et al*. reported that rats’ ability to discriminate *Mycobacterium* TB (*MTB*) from non-tuberculous *Mycobacterium* and other bacteria was by sniffing MTB-specific varieties of volatile organic compounds [5].

In the present study, while it was encouraging to find nearly half (48%) of the additional TB cases by using trained rats that otherwise were missed by the routine TB screening at the DOT facilities, there was a difference between smear (59%) and Xpert sites (44%). According to our data, the odds of finding additional TB cases were lesser by a quarter when switching from smear to Xpert settings, nonetheless the Xpert sites were likely to detect more case among TB patients with lower bacillary loads. On the other hand, the 44% additional TB case finding at the Xpert site is still considerable, given these cases would have been undiagnosed and untreated without the rats second-line TB screening. It is worth noting that rats would have contributed more if verification tests after the rat’s indication as positive had been verified by culture, which is a gold standard, and can detect TB with far lower concentrations of mycobacterium in a sample than concentrated ZN smear, or by molecular WHO-recommended rapid diagnostics, such as Xpert MTB/RIF Untra or Truenat. In line with the recent report by Agizew *et al*., rats’ contribution has implications for Tanzania’s efforts to eliminate TB, especially that those TB patients who were identified using rats were considered TB negative (missed TB cases) in the context of the WHO-recommended rapid diagnostic setting and would not have received treatment otherwise and continued transmission of TB in the community [9].

### TB detection using rats by *Mycobacterium* bacillary load between Xpert and smear sites

Comparing the effect of TB detection, rats showed a favorable advantage in identifying TB among patients with a lesser bacillary load (AFB 1 + or scanty) in Xpert than smear settings. This difference, however, was not that substantial or significant between children and adults. Among Xpert and smear testing sites, the above finding was not surprising since patients with higher bacillary loads had a higher chance of being picked by Xpert, which has higher sensitivity than that of smear, at DOT facilities. On the other hand, as reported previously [9,11], our data suggests that rats have the potential to indicate TB when the bacillary load is less than the level required for Xpert positivity. Recent studies have shown that Xpert performs poorly when used to detect TB among samples with paucibacillary or very low bacillary loads [14,15]. In our setting, the outcome might have been different if DOT facilities had used Xpert Ultra, which appears to be unaffected by very low bacillary load [16]. Xpert Ultra has eight-fold higher analytical sensitivity (16 colony-forming units per milliliter (cfu/ml) versus 131 cfu/ml) than the conventional Xpert [17]. Further research is required to determine the incremental value of rats when Xpert Ultra is used, particularly because of its increased sensitivity, which makes it the recommended primary TB diagnostic test [18].

### Rat’s sensitivity and specificity against TB cases with microbiological evidence by smear and Xpert

This study for the first time demonstrated that rat-based TB detection has an incremental value over Xpert. Furthermore, our research reveals that rats exhibited significantly higher sensitivity (16 times higher) in patients who tested positive by Xpert, compared to those who tested positive by smear in the sputum samples we use for rat-rewarding purposes. The specificity was also higher by 1.3 times between smear- and Xpert-negative presumed TB patients. This result was not unexpected, though. Supporting our sensitivity data, previous research has shown that, unlike rats and Xpert, smears may not be able to discriminate MTB and other ABF-positive rod-shaped bacteria that may cause lung disease, such as non-tuberculous mycobacteria, Rhodococcus equi, and Nocardia species [19]. While rat’s sensitivity against smear-positive patients was consistent with the previous meta-analysis (82.3% versus 81.3%) by Kanaan *et al* [7], it was encouraging to find out for the first time that rat’s sensitivity against Xpert-positive TB patients reached at 98.7%, suggesting the potential for the first-line TB screening test. Mulder *et al* and Beyene *et al* from Tanzania and Ethiopia, respectively, has also reported on rats’ sensitivity against Xpert and Xpert Ultra tests, which were 82% [8] and 88% [11], respectively. The latter was a case report among eight TB cases tested by Ultra in Addis Ababa, Ethiopia. In addition to the exhibited comparable sensitivity, using trained rats has the following benefits over the standard of care, smear, or Xpert-based algorithm: 1). High throughput, i.e., fast, trained rats can sniff 100 samples in just 20 minutes and indicate whether the samples are positive or negative for TB. [3,20], and 2). Cheaper with one USD cost per sample test as opposed to USD18 when using Xpert [3]. Therefore, APOPO’s efficient and cost-effective TB detection technology has the potential to contribute to TB elimination goals and global END TB strategies.

Re-evaluating (second-line TB screening) sputum samples tested by Xpert or smear among presumed TB patients is the current method used for rat-based TB detection [11,21]. Rat-based TB detection is a potential candidate for a novel, sustainable, first-line TB screening method, particularly in low- and middle-income countries where the risk of TB transmission in the community is high. The rats’ abilities to detect TB as a first-line TB screening technology have never been studied before. Evaluating rat-based TB diagnosis using a non-inferiority approach to compare them with the WHO-recommended molecular tests, Xpert/Ultra, warrants, given the above-mentioned advantages and comparable sensitivities against the rapid molecular tests and detection of paucibacillary TB, which Xpert might miss.

Our study has some limitations. First, for those samples evaluated by rats after the Xpert MTB/RIF negative test result, using the same sample for rats was not possible. Thus, a second sample was collected and evaluated by rats. In such cases, we cannot rule out the possibility of inter-sample variations, i.e., despite the negative result for the first sample, the second sample might have been positive if it was tested by Xpert MTB/RIF before rats’ evaluation. Second, the confirmatory test that we used after rat-positive indication was centrifuged and concentrated sample, which provided higher sensitivity than a routine direct smear method. However, if verification tests after the rat’s indication as positive had been verified by culture, which is a gold standard, or by molecular WHO-recommended rapid diagnostics, such as Xpert MTB/RIF Untra or Truenat, rats’ contribution would have been more than we just reported. Third, because the data were from a routine program setting, the age of certain TB cases was missing, which prevented them from being further analyzed. Fourth, health workers at various DOT facilities may not have received training before the data collection, and the TB screening and recording procedures may not have been consistent, which may result in inconsistent presumptive patient identification. Fifth, even though sputum induction is usual at DOT facilities, it was possible to collect insufficient sputum samples from children, which may have resulted in fewer pediatric TB diagnoses than in adult patients.

In conclusion, in our high-TB burden settings that use Xpert or smear-diagnostic algorithms, using trained rats led to the identification of one-third of the TB cases reported at DOT facilities. Though the odds of finding additional TB cases were lesser by a quarter when switching from smear to Xpert settings, it is noteworthy that the impact of rat-based TB detection was substantially greater in Xpert settings than that of smears among TB patients with a lower bacillary load. In the present study, rats proved to be crucial to the current TB control effort in Tanzania, even in the context of rapid molecular testing settings. Specifically, rats helped uncover TB cases that were missed in routine program diagnostic settings. Given that Xpert Ultra’s sensitivity is higher than Xpert’s limited ability to detect paucibacillary TB, further validation of this result using Xpert Ultra is recommended. Furthermore, as this has never been done before, we recommend further research to evaluate the rats’ abilities to detect TB using a non-inferiority approach, compared to the WHO-recommended rapid molecular tests (Xpert or Xpert Ultra), and determine whether rats may be used as a first-line TB screening method.

## Supporting information

S1 File Dataset_inrcemental value of rats over smear and Xpert. (XLSX)
